# Networks of Host Factors that Interact with NS1 Protein of Influenza A Virus

**DOI:** 10.3389/fmicb.2016.00654

**Published:** 2016-05-04

**Authors:** Sathya N. Thulasi Raman, Yan Zhou

**Affiliations:** ^1^Vaccine and Infectious Disease Organization – International Vaccine Centre, University of Saskatchewan, SaskatoonSK, Canada; ^2^Vaccinology and Immunotherapeutics Program, School of Public Health, University of Saskatchewan, SaskatoonSK, Canada

**Keywords:** swine influenza virus, NS1 protein, swine respiratory epithelial cells, strep-tag, protein interaction networks

## Abstract

Pigs are an important host of influenza A viruses due to their ability to generate reassortant viruses with pandemic potential. NS1 protein of influenza A viruses is a key virulence factor and a major antagonist of innate immune responses. It is also involved in enhancing viral mRNA translation and regulation of virus replication. Being a protein with pleiotropic functions, NS1 has a variety of cellular interaction partners. Hence, studies on swine influenza viruses (SIV) and identification of swine influenza NS1-interacting host proteins is of great interest. Here, we constructed a recombinant SIV carrying a Strep-tag in the NS1 protein and infected primary swine respiratory epithelial cells (SRECs) with this virus. The Strep-tag sequence in the NS1 protein enabled us to purify intact, the NS1 protein and its interacting protein complex specifically. We identified cellular proteins present in the purified complex by liquid chromatography-tandem mass spectrometry (LC-MS/MS) and generated a dataset of these proteins. 445 proteins were identified by LC-MS/MS and among them 192 proteins were selected by setting up a threshold based on MS parameters. The selected proteins were analyzed by bioinformatics and were categorized as belonging to different functional groups including translation, RNA processing, cytoskeleton, innate immunity, and apoptosis. Protein interaction networks were derived using these data and the NS1 interactions with some of the specific host factors were verified by immunoprecipitation. The novel proteins and the networks revealed in our study will be the potential candidates for targeted study of the molecular interaction of NS1 with host proteins, which will provide insights into the identification of new therapeutic targets to control influenza infection and disease pathogenesis.

## Introduction

Influenza A viruses are globally important human and animal pathogens responsible for seasonal, epidemic and periodically world-wide pandemic outbreaks. Among these viruses, swine influenza viruses (SIV) are a common and an important cause of respiratory disease in pigs (Choi et al., 2002; Palese, 2004). Pigs are an important host of influenza viruses which could harbor viruses with a pandemic potential (Hinshaw et al., 1981; Kida et al., 1994; Alenzi, 2010), as they are known to support the replication of both human and avian viruses. It has been shown experimentally that avian viruses of various HA subtypes, including those containing non-human HA subtypes can infect and replicate in pigs. Additionally, infection of pigs with a mixture of swine virus and an avian virus, which are unable to replicate in pigs, resulted in the generation of a reassortant that could replicate in pigs (Kida et al., 1994). The 1957 and 1968 pandemics were caused by reassortant viruses, believed to have been generated by mixing of different strains of viruses after infection in pigs (Kahn et al., 2014). The recent 2009 pandemic caused by a swine origin H1N1 virus was also generated by reassortment in pigs (Neumann et al., 2009). Hence, pigs serve as a mixing vessel for the generation of novel viruses with pandemic potential. Several cases of transmission of influenza from pigs to humans have also been documented, which sometimes result in fatal outcomes for humans (Hodder et al., 1977; Wells et al., 1991; Myers et al., 2007; Jhung et al., 2013). Thus, there is a need for a better understanding of SIV biology and the molecular mechanisms involved during infection. However, knowledge on the molecular basis of SIV infections is very limited and especially very little is known about the host-virus protein interactions critical during SIV infection.

Antiviral drugs form an important part of the arsenal for the fight against influenza virus infections. Currently, two classes of drugs are FDA-approved and available for treatment of influenza virus infection: M2 ion channel inhibitors (amantadine, rimantadine) and Neuraminidase inhibitors (NAIs) (oseltamivir, zanamivir) (Davies et al., 1964; Gubareva et al., 2000; Hayden, 2001). However, a major problem is the frequent emergence of viral resistance against these antiviral drugs. For example, usage of amantadine and rimantadine, collectively called as adamantanes, resulted in the rapid emergence of resistant strains that it is no longer recommended for influenza antiviral treatment (Bright et al., 2005). Resistance against oseltamivir, zanamivir and newly developed NAIs peramivir and laninamivir has also been reported (Hurt et al., 2009; Kamali and Holodniy, 2013; Orozovic et al., 2014; McKimm-Breschkin and Barrett, 2015). The error-prone nature of the viral RNA polymerase makes it easy for the virus to gain resistance to drugs targeting viral proteins (Watanabe et al., 2014). Therefore, nowadays there is a growing emphasis for developing drugs targeting host factors, where the possibility of the virus gaining drug-resistance is minimal (Lou et al., 2014). Many antiviral drugs targeting host factors are under different stages of development. One among them is the sialidase drug DAS181, which removes sialic acids on respiratory epithelial cells and prevents virus attachment to the receptor (Belser et al., 2007). Resistance to the drug was minimal and unstable upon extensive passaging of influenza virus in the presence of DAS181 (Triana-Baltzer et al., 2011). Other approaches using inhibitors to target cellular proteases (Bottcher-Friebertshauser et al., 2010, 2011) and the Raf/MEK/ERK signaling pathway (Droebner et al., 2011) have also been demonstrated to be successful in inhibiting virus infection. Treatment with immunomodulatory drugs targeting the host immune system, such as COX-2 inhibitors and S1P agonists have been found to alleviate tissue damage caused by virus-induced cytokines and also suppress virus replication (Lee et al., 2008, 2011; Marsolais et al., 2008). Additionally, many other inhibitors that target cellular factors are known to affect various stages of the influenza virus life cycle leading to reduced virus replication (Muller et al., 2012). Thus, identification of host factors affecting influenza virus infection is of paramount importance for the basic understanding of virus life cycle and can lead to novel antiviral therapy.

The NS1 protein of influenza A viruses is involved in regulating splicing, export and translation of viral mRNA, antagonizing host defenses and it fulfills these functions via interaction with multiple cellular partners (Hale et al., 2008; Marc, 2014). Although a variety of NS1 interaction partners have been identified, there is still a great interest in discovering novel interaction partners (Shapira et al., 2009; de Chassey et al., 2013). During infection, the primary target for influenza viruses are the respiratory epithelial cells in the trachea (Hers, 1966; Manicassamy et al., 2010). Therefore, studying SIV replication by infecting primary swine respiratory epithelial cells (SRECs) will resemble conditions close to natural infection. Additionally, when compared to well-established cell lines, analyzing protein interactions in primary cells may yield novel NS1-interacting cellular partners. Thus, in this study, we constructed a recombinant SIV carrying a Strep-tag in the NS1 protein in order to facilitate the isolation of an intact NS1-interacting protein complex from infected SRECs. We identified cellular proteins present in the purified complex by liquid chromatography-tandem mass spectrometry (LC-MS/MS) and generated a dataset of these proteins. We further analyzed the dataset by bioinformatics and provided comprehensive networks of host factors that interact with NS1. The interaction of several specific cellular proteins with NS1 was validated by immunoprecipitation.

## Materials and Methods

### Cells and Viruses

New born porcine tracheal epithelial (NPTr) cells, which are non-transformed and non-carcinoma SREC line capable of supporting efficient replication of influenza viruses (Ferrari et al., 2003; Delgado-Ortega et al., 2014; Thulasi Raman et al., 2016), were maintained in minimum essential medium (MEM) supplemented with 10% fetal bovine serum (FBS). Madin-Darby canine kidney (MDCK) cells were also maintained in MEM supplemented with 10% FBS. SRECs were isolated and cultured as described elsewhere (Yamaya et al., 1992). Briefly, trachea from healthy 6–8 week old pigs was obtained after euthanization and washed in sterile phosphate buffered saline (PBS). The trachea was transected longitudinally and the surface epithelium was pulled off the submucosa using a glass microscope slide. The epithelium was washed with Joklik’s MEM (JMEM) (Sigma–Aldrich) containing glutamine (2 mM), Dithiothreitol (0.5 mg/ml) (Sigma–Aldrich), Deoxyribonuclease (10 μg/ml) (Sigma–Aldrich) and antibiotics for 3 h at 4°C, changing the media every 1 h. The tissue was then digested with protease XIV (Sigma–Aldrich) in the above media for 18 h at 4°C. After 18 h, the protease XIV was neutralized by adding FBS to a final concentration of 20%. The media was filtered through a 70 μm cell strainer and the cells were pelleted down and washed with JMEM supplemented with 20% FBS. The cells were then resuspended in Bronchial Epithelial Growth Medium (BEGM) (Lonza Group Ltd.) with added growth factors from the bullet kit (Lonza Group Ltd.) and then plated on an uncoated dish for 2–3 h and left in an incubator at 37°C. Contaminating fibroblasts attach to the plate, while epithelial cells are only lightly attached and can be easily dislodged by gentle agitation. The floating cells were once again washed in BEGM media and plated onto a Type IV collagen (Sigma–Aldrich)-coated cell culture flask. Once the cells reached 100% confluency, they were split on to Type IV collagen-coated flasks and propagated further.

A/Sw/SK/18789/02 (H1N1) (SK02) virus was propagated in 11-day-old embryonated chicken eggs as described previously (Shin et al., 2007a). Influenza virus titer was determined by plaque assay on MDCK cells.

### Antibodies and Reagents

Rabbit polyclonal anti-NS1 and NP antibodies were generated in our laboratory as previously described (Shin et al., 2007b). The other antibodies were purchased from different sources as follows: Strep MAB-Classic antibody conjugated to horseradish peroxidise (HRP) (IBA), Alkaline Phosphatase (AP) – Conjugated anti-rabbit IgG (Jackson ImmunoResearch), Mouse anti-Flag M2 antibody (Sigma–Aldrich), Rabbit anti-flag DYKDDDDK tag antibody (Cell signaling technology), IRDye 680RD anti-Rabbit antibody (LI-COR), Mouse anti-human cytokeratin (pan) (AbD Serotec), Goat F(ab’)2 Anti-Mouse IgG1- FITC conjugate (Southern Biotech), mouse IgG1 negative control antibody (AbD Serotec), Goat anti-Rabbit IgG secondary antibody, Alexa Fluor 594 (Life Technologies).

Other reagents were purchased from the following sources as follows: TransIT-LT1 (Mirus Bio LLC), Strep-Tactin Sepharose 50% suspension (IBA), Strep-tag II peptide (IBA), D-Desthiobiotin (IBA), Dynabeads protein G (Life Technologies), Ni-NTA agarose resin (Qiagen).

### Plasmid Construction and Generation of Mutant Viruses

The DNA sequence corresponding to the Strep-tag (TGGTCACACCCACAGTTCGAAAAA) was introduced into pHW-SK-NS (Masic et al., 2009) plasmid by overlapping PCR. Plasmid pHW-SK-NS-Strep-replacement encodes SIV/SK-NS1 with amino acids (AA) 77-84 replaced by the Strep-tag, while plasmid pHW-SK-NS-Strep-insertion encodes SIV/SK-NS1 with the Strep-tag inserted between AA 79 and 80.

The recombinant viruses encoding the Strep-tag NS1 were rescued using an eight-plasmid reverse genetics system (Hoffmann et al., 2002). For rescue of the recombinant viruses, plasmids pHW-SK-PB2, pHW-SK-PB1, pHW-SK-PA, pHW-SK-HA, pHW-SK-NP, pHW-SK-NA, pHW-SK-M (Masic et al., 2009) and one of either plasmids pHW-SK-NS-Strep-replacement or pHW-SK-NS-Strep-insertion were transfected onto co-cultured MDCK and 293T cells. The rescued viruses designated SIV/SK-544 and SIV/SK-545 were propagated in 11-day-old embryonated eggs.

### Western Blotting

A portion of the cell lysate (input), eluent or wash fractions from the Strep-tactin sepharose pull-down experiment, immunoprecipitation (IP) and samples for protein expression kinetics were resolved by sodium dodecyl sulfate – 10% polyacrylamide gel electrophoresis (SDS-PAGE) and Western blotting was performed as described elsewhere (Shin et al., 2007b).

### Real-time PCR

To measure IFNβ mRNA level in virus infected cells, RNA was extracted from cells using the RNeasy mini kit. 500 ng of extracted RNA was used for reverse transcription using oligo (dT)_20_ primer and SuperScript III reverse transcriptase to generate cDNA. The cDNA was then combined with the primer set and SYBR green super mix. Quantitative real-time PCR (qPCR) reaction was performed in an iCycler IQ5 multicolor real-time PCR detection system (Bio-Rad, Hercules, CA) using the following primers: porcine IFNβ forward, 5′-CCGAATTCGCTAACAAGTGCATCCTCC-3′; porcine IFNβ reverse, 5′-GCGAAGCTTTCAGTTCCGGAGGTAATC-3′; porcine RPL19 forward, 5′-AACTCCCGTCAGCAGATCC-3′; porcine RPL19 reverse, 5′-AGTACCCTTCCGCTTACCG-3′. RPL19 gene transcription was used for normalization of IFNβ expression. Data are presented as relative gene expression to that of mock infected cells using the formula 2^-ΔΔ^*^Ct^* (Livak and Schmittgen, 2001). Real-time PCR was conducted in triplicate for each sample and the mean value was calculated. Final figures represent the results from three independent experiments.

### Purification of Strep-tagged NS1 Protein Complex and Identification of Interacting Proteins

SRECs’ were infected with SIV/SK-544 at an MOI of 2. At 16 h post infection (h.p.i.), cells were harvested in cell lysis buffer (Cell Signaling Technology) with protease inhibitor (Complete protease inhibitor cocktail tablets – Roche Diagnostics Corporation). The lysate was sonicated and then clarified by centrifugation at 16200 × *g* for 10 min at 4°C. The Strep-tactin sepharose resin (IBA) was washed three times with four column volume (CV) of cell lysis buffer and then was added to the clarified lysate and incubated at 4°C overnight. Next, the lysate-sepharose mixture was added to a polypropylene column (Qiagen) and the sepharose was washed extensively with wash buffer [100 mM Tris-Cl (pH 8.0), 150 mM NaCl, 1 mM EDTA]. The Strep-tag NS1 protein complex was then eluted from the sepharose resin with 3 CV of elution buffer (wash buffer with 2.5 mM desthibiotin). Protease inhibitor cocktail was added to the eluent to prevent degradation of the proteins and the proteins present in the complex were identified by LC-MS/MS at the University of Victoria-Genome BC Proteomics Centre.

### Analysis of MS Data

The proteins identified by LC-MS/MS were enriched and grouped into different functional categories using the functional annotation tool of DAVID Bioinformatics Resources 6.7 (Jiao et al., 2012). The proteins from our NS1-interacting complex were screened against the known NS1-host factor interactions from VirHostNet (Virus–Host Network) 2.0, which is a public knowledgebase dedicated to the management, analysis and integration of virus–host interactions (Navratil et al., 2009; Guirimand et al., 2015). Proteins annotated to be involved in innate immunity were screened using the Gene Ontology Analysis tool from InnateDB database (Lynn et al., 2008; Lynn et al., 2010; Breuer et al., 2013).

### Construction of Protein–Protein Interaction Networks

Interaction networks of the proteins were generated through the use of STRING database (version 10) (Jensen et al., 2009) and Cytoscape software (version 3.2.0) (Shannon et al., 2003).

### Transfection and IP

To examine NS1 interaction with the His-tagged and Flag-tagged cellular proteins, 293T cells were seeded at a density of 1 × 10^6^ cells/well in six-well plates. One micro gram of each plasmid encoding the protein of interest was transfected using TransIT-LT1 as per the manufacturer’s recommendation. The plasmids used for transfection include pcDNA-PR8-NS1, pCMV-3 × Flag-hnRNP C, pCDNA4-HisMaxA-hnRNP K, pCDNA4-HisMaxA-hnRNP U, pCDNA4-HisMaxA-hnRNP F, pCDNA4-HisMaxA-hnRNP M, pCDNA4-HisMaxA-hnRNP R, pcDNA4-HisMaxA-ILF2, pCDNA4-HisMaxA-ILF3, pCDNA4-HisMaxC-DDX1. These cellular proteins were cloned into pCMV-3 × Flag, pcDNA4-HisMaxA and pCDNA4-HisMaxC plasmids, such that the Flag- or His-tag is fused at the N-terminal of the expressed fusion protein.

For examining protein interaction between Flag-hnRNPC and NS1 by IP, the cell lysate was collected in 1 ml Flag lysis buffer (FLB) (50 mM Tris HCl, pH 7.4, with 150 mM NaCl, 1 mM EDTA, and 1% TRITON X-100). The cell lysate was then sonicated and cleared of cellular debris by centrifugation. Then, 1.5 μg mouse monoclonal anti-Flag M2 (Sigma–Aldrich) antibody was added to the cell lysate and was incubated with gentle rocking at 4°C for 1 h and 15 min. Next, 35 μl of Dynabeads Protein G (Life Technologies) was added to the lysate and was further incubated for another 1 h and 15 min with gentle rocking at 4°C. The beads were then washed extensively with FLB and the precipitated proteins were subjected to Western blotting with appropriate antibodies.

For examining protein interaction between the His-tagged cellular proteins and NS1, the cell lysate was collected in 1 × cell lysis buffer (Cell Signaling Technology). The Ni-NTA agarose resin (Qiagen) was washed in PBS and was incubated with the lysate for 2 h at 4°C. The beads were then washed three times with FLB and three times with PBS. The precipitated proteins were subjected to Western blotting with appropriate antibodies.

### Statistical Analysis

The statistical significance of differences was calculated using GraphPad Prism 6 (GraphPad Software, Inc., USA) with parametric *t*-tests to obtain the *P*-value. Data are shown as mean ± SD (standard deviation) of three independent experiments. Significant differences between treatments and controls are represented by an asterisk (*P* < 0.05) or two asterisks (*P* < 0.01).

## Results

### Construction and Characterization of Strep-tagged NS1 Mutants

**FIGURE 1:**
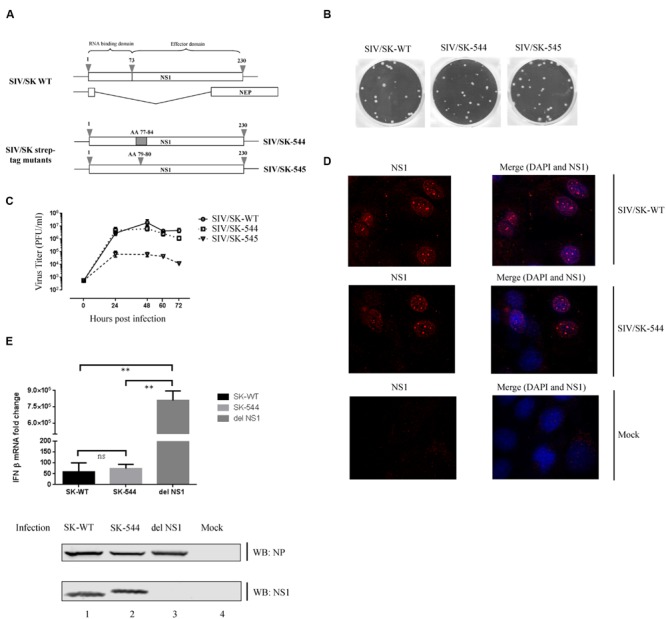
**Characterization of recombinant Strep-tag NS1 viruses. (A)** A schematic representation of the NS1 protein and the location of the Strep-tag replacement and insertion within the NS1 protein sequence. **(B)** MDCK cells were infected with either the WT virus or the recombinant Strep-tag viruses. At 48 h.p.i., plaques were visualized by staining with coomassie blue. **(C)** MDCK cells were infected with either the WT virus or the recombinant Strep-tag viruses at an MOI of 0.001 in triplicates. Supernatant was collected every 24 h and the titer was determined by plaque assay on MDCK cells. A growth curve was plotted using the mean titer values at each time point and the associated standard deviation is displayed as error bar. **(D)** MDCK cells were infected with either the WT virus or the mutant SIV-SK/544 virus at an MOI of 2. At 8 h.p.i., the cells were fixed, permeabilized and stained with rabbit anti-NS1 serum followed by Alexa Fluor 594-conjugated anti-rabbit antibody (red) and mounted with a mounting media containing DAPI stain (blue). A representative image of multiple fields of view is shown. **(E)** NPTr cells were infected with SIV/SK-WT, SIV/SK-544, or PR8 del NS1 virus at an MOI of 1. At 12 h.p.i., the cell lysate was collected and RNA was extracted. The RNA was reverse transcribed and the cDNA was then used in qPCR experiments to determine IFNβ mRNA expression. Real-time PCR was conducted in triplicate for each sample and the mean value was calculated. Final figures represent the results from three independent experiments and the associated standard deviation is displayed as an error bar. Significant differences between treatments and controls are represented by an asterisk (*p* < 0.05) or two asterisks (*p* < 0.01). Samples with *p* < 0.05 were considered statistically significant, while *p* > 0.05 was considered not significant (ns). The expression of viral proteins present in the infected cell lysate was detected by Western blotting.

The NS1 protein is made of an RNA binding domain and an effector domain joined by a flexible and highly variable linker region (Qian et al., 1994; Bornholdt and Prasad, 2008). Our lab had previously rescued mutant viruses having a TC-tag insertion or a Strep-tag insertion in this variable region of NS1 (Li et al., 2010; Lin et al., 2012). Since we were interested in studying SIV NS1-interacting partners in its natural host, we rescued a recombinant SIV possessing a Strep-tag sequence (WSHPQFEK) in the linker region of NS1 protein in the background of SK02 virus. The presence of the Strep-tag sequence would enable the purification of NS1 protein along with any associated proteins from the infected cell lysate. Two mutant viruses were rescued: SIV/SK-544 that encodes NS1 with a Strep-tag sequence replacing AA 77 to 84 and SIV/SK-545 that encodes NS1 with a Strep-tag sequence inserted between AA 79 and 80 (**Figure 1A**).

The replication potential of the recombinant viruses was first assessed by comparing the plaque size and multiple cycle growth kinetics to that of the wild type (WT) virus. Even though plaque size of both mutants appear to be similar to WT virus (**Figure 1B**), SIV/SK-545 was attenuated in multi-cycle growth kinetics, while SIV/SK-544 displayed growth kinetics similar to WT virus (**Figure 1C**).

We further characterized the phenotype of the mutant virus to ensure that the introduction of the Strep-tag sequence did not alter the function of the NS1 protein. Since SIV/SK-544 mutant virus displayed similar plaque size and growth kinetics as the WT virus, we focused on the SIV/SK-544 mutant for further characterization. Influenza NS1 has been shown to predominantly localize in the nucleus of infected cells (Greenspan et al., 1988; Newby et al., 2007) and its intracellular distribution is vital for its multifunctional ability. Therefore, we immunostained WT and SIV/SK-544 virus-infected cells with antibodies specific for NS1 and observed the intracellular localization of NS1 protein in the infected cells at 8 h.p.i. As shown in **Figure 1D**, NS1 localized to the nucleus and nucleolus in both SIV/SK-544 and WT virus-infected cells, revealing a similar distribution pattern.

It is widely recognized that a major function of influenza virus NS1 is to antagonize the host type-I interferon (IFN) response (Hale et al., 2008; Marc, 2014; Ayllon and Garcia-Sastre, 2015). Influenza viruses devoid of NS1 protein expression have been instrumental in studying this response and have been observed to be attenuated, inducing large amounts of IFN in infected cells (Egorov et al., 1998; Garcia-Sastre et al., 1998; Haye et al., 2009). Therefore, we compared the ability of SIV/SK-544 and SIV/SK-WT viruses to inhibit the IFNβ response by measuring IFNβ mRNA levels using quantitative real-time PCR (q-PCR) in infected cells. A PR8 virus lacking the NS1 gene (del NS1 virus) was used as a positive control for IFNβ expression. As shown in **Figure 1E**, the IFNβ mRNA expression levels were reduced by 14000-fold and 11000-fold in SIV/SK-WT and SIV/SK-544 infected cells, when compared to IFNβ mRNA expression in del NS1 virus infected cells. Western blotting analysis of viral NP and NS1 proteins indicated that SIV/SK-WT and SIV/SK-544 expressed similar levels of NP and NS1 at 12 h.p.i. (lane 1 and 2), while only NP protein expression was observed in del NS1 virus infected cell lysate (lane 3). These results show that introduction of the Strep-tag sequence does not affect NS1 expression, localization and function of the recombinant virus. Therefore, we used SIV/SK-544 virus for further studies to isolate the intact NS1-interacting protein complex from infected cells.

### Identification of Cellular Interaction Partners of NS1 Protein from Infected Primary SRECs

**FIGURE 2:**
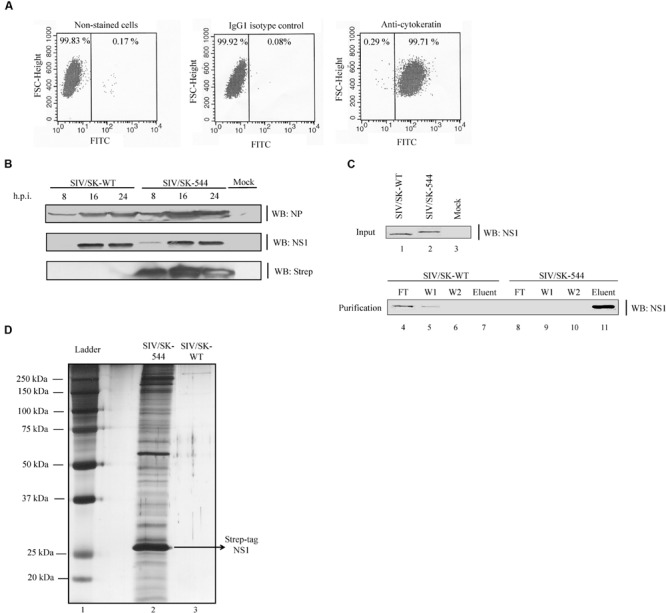
**Protein expression kinetics and NS1 protein Strep-tactin pull-down from WT and SIV/SK-544 virus infected SRECs. (A)** SRECs were stained with an antibody against the epithelial cell marker cytokeratin and the purity of the cells was analyzed using a fluorescence-activated cell sorter machine. **(B)** SRECs were infected with WT or SIV/SK-544 virus at an MOI of 1. Cell lysate collected at the indicated time points were subjected to Western blotting with antibodies specific for NP, NS1, and Strep-tag. **(C)** SRECs were infected with either the WT virus or SIV/SK-544 virus at an MOI of 2. At 16 h.p.i., cell lysate was collected and subjected to a pull-down assay with Strep-tactin sepharose. The Flow through (FT), Wash fractions (W1, W2) and the Eluent were collected and subjected to Western blotting with antibody against NS1. **(D)** The eluent from the SIV/SK-544 and SIV/SK-WT lysate were run on a SDS-PAGE gel and the proteins present in the eluent were visualized by silver staining.

To identify the interactors in a more relevant natural infection setting, primary SRECs were isolated from the trachea of healthy 6–8 week old pigs and cultured on collagen-coated culture flasks. The cultured primary cells displayed typical cobble-stone morphology (data not shown) and almost all of the cultured cells stained positive for the epithelial cell marker cytokeratin (Bateman et al., 2013) (**Figure 2A**), confirming their epithelial nature.

To ensure that the virus can replicate efficiently in primary SRECs, we infected SRECs with the WT and mutant viruses at an MOI of 1 and collected the cell lysate at different time points. The cell lysate was then subjected to Western blotting with antibodies against NS1, NP and Strep-tag. The protein expression kinetics was found to be similar for both WT virus and SIV/SK-544 virus in infected SRECs (**Figure 2B**). Moreover, while the NS1 antibody detected both WT NS1 and Strep-tagged NS1, only Strep-tagged NS1 was detected by the antibody against Strep-tag.

We next tested the accessibility of the Strep-tag to Strep-tactin, a derivative of streptavidin which binds to the Strep-tag specifically with high affinity (Schmidt and Skerra, 2007). SRECs were infected with either WT or SIV/SK-544 virus. At 16 h.p.i., the cell lysate was prepared and subjected to a pull-down assay with Strep-tactin resin. We chose the 16 h time point for cell lysate collection as the NS1 protein expression in SIV/SK-544 SRECs was robust at this time point, when compared to 8 h.p.i. (**Figure 2B**). To identify viral and cellular proteins that may bind non-specifically to the Strep-tag alone, purified Strep-tag peptide was added to the WT virus-infected lysate and incubated along with the Strep-tactin resin in the pull-down experiment. The presence of NS1 in the eluent, flow-through and wash fractions was determined by Western blotting (**Figure 2C**). Only NS1 from SIV/SK-544-infected cells could be pulled down efficiently (lane 11), while the WT NS1 protein did not bind to the resin and was lost during the washes (lanes 4 and 5). The eluent from WT virus-infected and SIV/SK-544-infected cell lysate were also analyzed by SDS-PAGE and silver staining (**Figure 2D**). The band corresponding to NS1 in SIV/SK-544 eluent was visible in the silver-stained gel and proteins interacting with NS1 were also co-eluted (lane 2). In contrast, no visible bands were observed in the eluent from WT virus-infected sample (lane 3).

The proteins present in the NS1 pull-down complex from SIV/SK-544- and WT virus-infected cells were then identified by LC-MS/MS. As a result, only five proteins were identified in the SIV/SK-WT eluent, whereas 445 proteins were identified in the eluent from SIV/SK-544-infected cells. While no viral proteins were identified in the SIV/SK-WT eluent, viral proteins PB2, PB1, PA, HA, NP, and M1 were identified in SIV/SK-544 eluent. The proteins present in the complex were identified using a software package called Mascot from Matrix Science ^1^, which compares the observed mass spectra data to a database of known proteins to determine the most likely matches. As the availability of swine protein sequences in the database is limited, the LC-MS/MS data was used to search against all the available mammalian sequences for protein identification. For each protein match, Mascot calculates an overall protein score, which reflects the combined score of all the observed mass spectra that can be matched to amino acid sequences within that protein. Therefore, a higher score indicates a more confident match. To be more stringent in specificity, we set a threshold to be a MASCOT protein score of 150 and above. Additionally, we also excluded those proteins identified with a single peptide match by LC-MS/MS and those proteins identified to be present in the SIV/SK-WT eluent. By setting up this threshold and exclusion, we were able to restrict our analysis to the proteins with the highest confidence scores and thereby ended up with 192 proteins (43% of the total number, Supplementary Table S1) for further analysis.

### Bioinformatics Analysis of the Protein Dataset

VirHostNet 2.0 database provides a high-confidence resource of manually curated interactions defined for a wide range of viral species including influenza virus. We screened the influenza virus–host interaction database in VirHostNet 2.0 against the 192 top scoring proteins identified in our MS analysis and found that 92 out of the 192 proteins have been documented in the VirHostNet 2.0 database as NS1-interacting partners (Supplementary Table S1). This analysis gave further proof that the proteins identified in our study are valid NS1 protein interaction partners.

NS1 exerts its multifunctional nature primarily by interacting with host proteins. Therefore, categorizing and grouping the proteins identified by LC-MS/MS on the basis of their molecular functions in the host will reveal potential pathways that might be affected by NS1 expression during infection. Gene Ontology enrichment analysis was performed using the DAVID bioinformatics resources (Jiao et al., 2012). A number of important terms were identified to be enriched including protein translation, RNA processing and splicing, microtubules and cytoskeleton and apoptosis (**Table 1**). These are known to be important host functions regulated during influenza virus infection and are discussed below.

**Table 1:** Table showing the DAVID functional annotation summary of the NS1-interacting protein dataset.

David functional annotation summary		
**GO term**	**No. of genes involved**	***P*-value**

Translation	59	4.80E–56
RNA processing	41	2.60E–21
RNA splicing	24	7.40E–13
Microtubule & Cytoskeleton	20	1.60E–04
Regulation of apoptosis	13	1.20E–01
PDZ	3	3.80E–01

*The number of genes involved in each of the enriched Gene Ontology (GO) term and the *P*-values corresponding to each term is listed.*

There are several reports supporting a role for NS1 in the selective translation of viral mRNAs over cellular mRNAs (Garfinkel and Katze, 1993; de la Luna et al., 1995; Park and Katze, 1995; Marc et al., 2013). It is widely believed that NS1 interacts with the 5′UTR of viral mRNA, eIF4GI, and PABP1 and facilitates recruitment of the 40S ribosomal subunit bound to eIF3, thereby enhancing selective translation of viral mRNA (Hale et al., 2008; Marc, 2014). NS1 protein has also been shown to interact with hStaufen (STAU1) (Falcon et al., 1999), which is known to contribute toward microtubular transport of cellular mRNAs to polysomes. This interaction is thought to enhance the selective translation of viral mRNA by NS1. In line with these observations, PABP1 and STAU1 were identified in the NS1-interacting complex in our study (Supplementary Table S1) along with several other cellular proteins involved in translation (Supplementary Table S2).

Once the vRNP is in the cytoplasm, it is transported into the nucleus by the nuclear import machinery in association with viral proteins (Wang et al., 1997; Cros et al., 2005). Once in the nucleus, genomic replication, transcription and pre-mRNA processing takes place. Influenza viruses are unique among RNA viruses in that the whole replication cycle takes place inside the nucleus and not in the cytoplasm (Herz et al., 1981), as the virus needs the host splicing machinery to splice the NS1 and M1 mRNA into smaller NS2 and M2 mRNA, respectively, (Lamb and Lai, 1980; Lamb et al., 1981). NS1 protein affects several functions in RNA processing. Influenza NS1 is reported to increase the splicing of viral M mRNA but does not appear to affect the splicing of its own mRNA (Robb et al., 2010; Robb and Fodor, 2012). Contrary to this enhancing effect, influenza NS1 protein is known to inhibit cellular pre-mRNA splicing by associating with spliceosomes and U6 snRNA, contributing to host’s shut-off (Lu et al., 1994). Moreover, influenza NS1 protein is also proposed to have a function in nuclear export of viral mRNAs and has been shown to interact with several proteins involved in nuclear export including NXF1 (Satterly et al., 2007). Its binding to cellular protein CPSF inhibits polyadenylation of host pre-mRNAs and prevents their nuclear export (Nemeroff et al., 1998). Various hnRNP proteins and the NXF1 interacting protein THOC4 are also involved in cellular mRNA splicing and processing (Dreyfuss et al., 1993; Masuda et al., 2005; Hautbergue et al., 2008). Thus, many of the proteins involved in RNA processing and splicing were identified in our study. Among the NS1-interacting host proteins, 41 proteins and 24 proteins were annotated by DAVID to be involved in RNA processing and RNA splicing, respectively, which includes CPSF1, CPSF2, 8 out of the 20 known hnRNP proteins and THOC4 (Supplementary Table S2).

**FIGURE 3:**
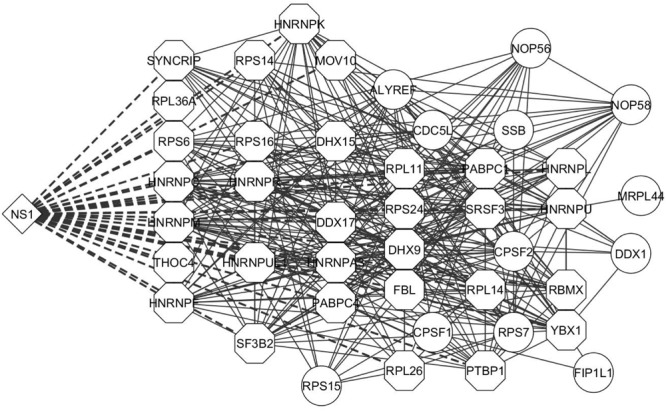
**Interaction network of proteins involved in RNA processing.** The proteins classified as related to RNA processing machinery were analyzed by STRING database and an interaction network was generated using Cytoscape. The edges connecting NS1 and known NS1-interacting host proteins are represented by dashed lines and the NS1-interacting host protein nodes are highlighted by an octagon shaped border. Nodes represent the proteins in the network. Edges represent the interaction connecting two nodes.

**FIGURE 4:**
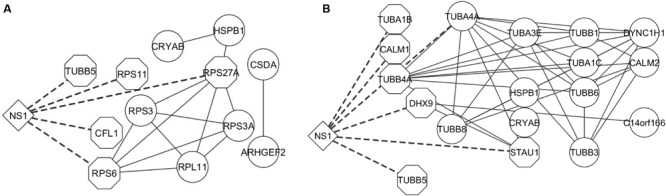
**Interaction network of proteins involved in Apoptosis and Cytoskeleton network.** The proteins classified as belonging to the **(A)** apoptosis pathway and **(B)** cytoskeleton network were analyzed by STRING database and an interaction network was generated using Cytoscape. The edges connecting NS1 and known NS1-interacting host proteins are represented by dashed lines and the NS1-interacting host protein nodes are highlighted by an octagon shaped border. Nodes represent the proteins in the network. Edges represent the interaction connecting the two nodes.

Apoptosis is an important innate immune mechanism to maintain homeostasis in the host and is a major pathway regulated during influenza infection (Takizawa et al., 1993; Hinshaw et al., 1994). Influenza virus has been documented to modulate apoptosis to favor efficient virus replication by interacting with several host proteins through its viral proteins including NS1 (Herold et al., 2012; Iwai et al., 2013). A recent study showed that NS1 interaction with β-tubulin disrupts the cellular microtubule network and thereby commits the cell to apoptosis (Han et al., 2012). NS1 interaction with the p85 regulatory subunit and CRK/CRKL prevents premature cell-death and facilitates enhanced virus replication (Shin et al., 2007c; Ehrhardt and Ludwig, 2009; Hrincius et al., 2010). Additionally, the microtubule and cytoskeleton network also contributes to trafficking of vRNPs from the cytoplasm to the cell periphery for assembly and budding of the virus (Amorim et al., 2011). Twenty and 13 proteins from our dataset were annotated by DAVID to belong to the microtubule and cytoskeleton network and apoptosis pathway, respectively, (Supplementary Table S2). Thus, the functions enriched by DAVID gene ontology analysis are relevant to the functions regulated by NS1 during virus infection and the NS1-interacting host proteins identified in our study could have important role in regulating these functions. Generation of interaction networks representing the proteins involved in RNA processing, apoptosis, and microtubule and cytoskeleton emphasized the close relationship between these factors to form a functional protein complex (**Figures 3** and **4A,B**, respectively).

**FIGURE 5:**
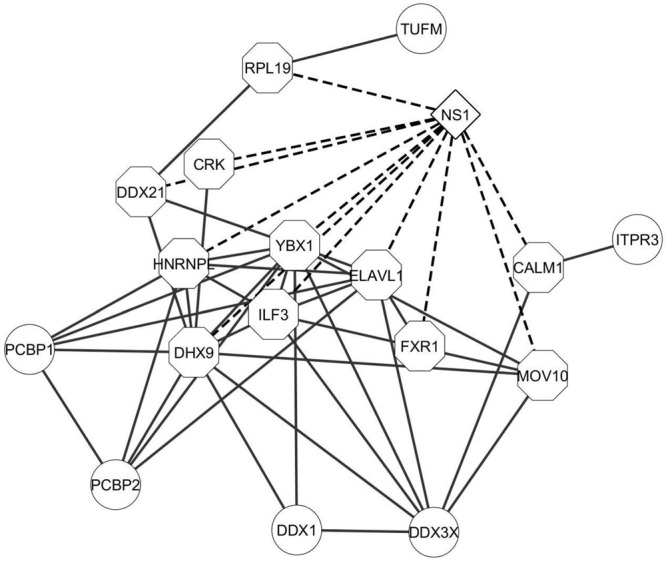
**Interaction network of innate immunity related proteins.** The proteins classified as belonging to innate immune response pathway were analyzed by STRING database and an interaction network was generated using Cytoscape. The edges connecting NS1 and known NS1-interacting host proteins are represented by dashed lines and the NS1-interacting host protein nodes are highlighted by an octagon shaped border. Nodes represent the proteins in the network. Edges represent the interaction connecting the two nodes.

NS1 is a known antagonist of innate immune response (Hale et al., 2008; Marc, 2014; Ayllon and Garcia-Sastre, 2015). Therefore, we analyzed the protein dataset for factors involved in innate immunity. InnateDB is a manually curated knowledgebase of the genes, proteins, interactions, and signaling responses in mammalian innate immunity (Breuer et al., 2013). Thus, we screened our dataset against the genes present in the innateDB database, which have been annotated to have a role in innate immune response. Eighteen proteins from our dataset were identified which belonged to this database (Supplementary Table S2). Several proteins listed in the table are well known interactors of NS1. Our laboratory and others reported that DHX9 and DDX21 interact with NS1 and regulate virus replication (Lin et al., 2012; Chen et al., 2014). Very recently, we also showed that DDX3 interacts with NS1 and plays an antiviral role through regulation of stress granule formation (Thulasi Raman et al., 2016). CRKL was reported to interact with NS1 and induce enhanced PI3K signaling (Ylosmaki et al., 2015). Other proteins such as FXR1 and ILF3 have known function during influenza infection, but have not been reported to interact with NS1 (Wang et al., 2009; Zhou et al., 2014). Hence, NS1 being an antagonist of innate immune responses, it might be interesting to study the interaction of these cellular proteins with NS1 and their function. A protein interaction network of these host factors revealed strong associations between these proteins (**Figure 5**).

### Validation of Specific Proteins in the NS1 Host Protein Interactors Dataset

**FIGURE 6:**
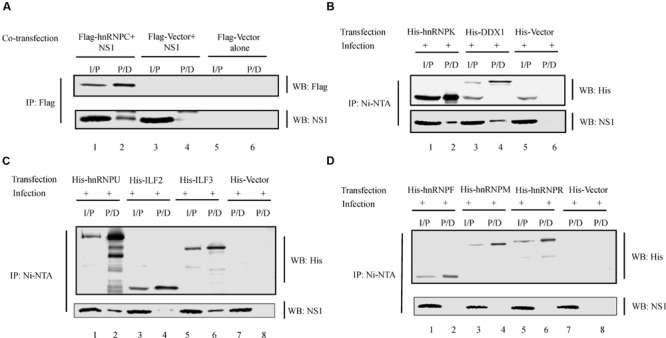
**NS1 interaction with specific cellular proteins. (A)** pcDNA-NS1 was co-transfected with plasmid encoding Flag-hnRNPC or Flag-vector in 293T cells. At 48 h.p. transfection, cell lysate was collected and subjected to IP with anti-Flag antibody. Precipitated proteins were subjected to Western blotting with antibodies against Flag-tag and NS1. **(B–D)** Plasmid encoding His-tagged hnRNPs or DDX1 was transfected in 293T cells. At 36 h.p. transfection, cells were infected with SIV/SK-WT at an MOI of 1. At 12 h.p.i., cell lysate was collected and subjected to IP with Ni-NTA agarose resin. Precipitated proteins were subjected to Western blotting with antibodies against His-tag and NS1. I/P = input. P/D = Pull-down.

hnRNP proteins are abundant nuclear proteins known to be bound to pre-mRNAs in the nucleus (Dreyfuss et al., 1993) and they function in splicing and nuclear export of pre-mRNA in eukaryotic cells (Dreyfuss et al., 1993; Chou et al., 1999), which are also functions shared by influenza virus premRNAs (York and Fodor, 2013). Multiple hnRNP proteins have been found to interact with viral proteins including hnRNP A2/B1 (Wang et al., 2014), hnRNP M and H1 (Jorba et al., 2008), and hnRNP A1 (Mayer et al., 2007). ILF3 is a dsRNA binding protein and has been shown to affect influenza virus replication by interacting with NP (Saunders and Barber, 2003; Wang et al., 2009; Wen et al., 2014). ILF2 is a binding partner of ILF3 and the ILF2-ILF3 heterodimer is involved in the post-transcriptional regulation of many genes in vertebrates (Jayachandran et al., 2016). Therefore, we tested the ability of several hnRNP proteins, ILF2, ILF3, and DDX1 present in our dataset to interact with NS1 protein. In order to test interaction between hnRNP C and NS1, plasmids encoding Flag-tagged hnRNP C and influenza virus NS1 proteins were co-transfected in 293T cells and the cell lysate was subjected to IP using anti-flag antibody (**Figure 6A**) (Note that Flag beads bind non-specifically to NP. Therefore, IP was not performed in infected cells to avoid non-specific pull-down of NS1 due to NP binding). 293T cells were transfected with plasmids encoding His-tagged hnRNP proteins hnRNP F, K, M, R, U, and ILF2, ILF3, and DDX1. The cells were then infected with SIV/SK-WT virus and the cell lysate was subjected to IP using Ni-NTA agarose (**Figures 6B–D**). The His- or Flag-tagged proteins and the NS1 protein present in the pull-down (P/D) were detected by Western blotting. While hnRNPs C, K, U, ILF3, and DDX1 co-precipitated NS1 (lower panels, lane 2 of **Figures 6A–C**, lane 6 of **Figure 6C**, lane 4 of **Figure 6B**), hnRNP F, M, R, and ILF2 failed to co-precipitate NS1 (lower panels, lanes 2, 4, 6 of **Figure 6D** and lane 4 of **Figure 6C**).

## Discussion

The NS1 protein is a major virulence factor of influenza A viruses and exerts its multifunctional nature by interacting with several cellular proteins (Hale et al., 2008; Marc, 2014). Several studies have used varied strategies to identify the NS1-interacting partners, which have advantages as well as limitations. While, yeast two-hybrid assay system is useful in studying dual interactions and has been used to identify novel host interaction partners of NS1 protein (Shapira et al., 2009; de Chassey et al., 2013), they do not represent interactions that occur in the context of virus infection or in protein complexes. Some studies identified cellular interaction partners of NS1 by exogenous expression of the viral protein in established cell lines and then co-precipitating the interacting cellular proteins by immunoprecipitation (Pichlmair et al., 2012; Watanabe et al., 2014). While this approach is useful in studying virus–host protein interactions that occur in a protein complex, it does not represent interactions that arise in the context of virus infection. Recently, a novel approach was used to rescue recombinant influenza viruses expressing epitope-tagged NS1 protein (Tawaratsumida et al., 2014). Even though, this approach resulted in the identification of physiologically relevant interactions that occur in the infected cells, both NS1 and NS2 proteins were tagged, which led to the inability to distinguish between proteins that interact specifically with either NS1 or NS2.

In our study, a strategy was devised to introduce a Strep-tag sequence specifically in the NS1 protein in order to analyze protein interactions of NS1 occurring during virus replication. The flexible and variable nature of the linker region between the RNA-binding domain (RD) and effector domain (ED) of NS1 has been demonstrated in several studies. Large scale sequence analysis of the NS1 sequence from various influenza A virus strains showed that the amino acid residues D74-L77 and K79-R83 in the linker region between the RD and ED of NS1 is highly variable (Darapaneni et al., 2009). Moreover, an X-ray structure study on NS1 observed the region corresponding to residues 75–79 as not well-defined, which shows that the linker region is highly flexible in nature (Bornholdt and Prasad, 2008). This region is amenable for modifications, which was demonstrated by previous studies in our laboratory involving tetracysteine (TC) tag and Strep-tag insertions in NS1 (Li et al., 2010; Lin et al., 2012). Taking advantage of this knowledge, an eight amino acid Strep-tag sequence was inserted in the SK02 NS1 linker region and a recombinant SK02 virus encoding a Strep-tag NS1 (SIV/SK-544) with growth properties, protein expression kinetics and anti-interferon function similar to that of WT virus was successfully rescued (**Figure 1**). This resulted in the introduction of the Strep-tag sequence specifically in NS1 without affecting the packaging signals or splicing of NS gene segment.

The Strep-tag sequence is engineered to bind with high affinity and reversibly to strep-tactin, enabling the isolation of intact protein complexes (Schmidt and Skerra, 2007). While previous studies utilized well-established cell lines for virus–host protein interaction studies, we performed our studies on primary cells isolated from the trachea of healthy pigs to resemble natural infection conditions. Thus, SRECs were infected with SIV/SK-544 and the presence of Strep-tag sequence in the NS1 protein facilitated the purification of an intact NS1-interacting protein complex (**Figures 2C,D**). The host and viral proteins present in the purified NS1–host protein complex were identified by LC-MS/MS. Since NS1 interaction with cellular proteins is critical for its multifunctional nature, the host proteins present in the purified complex could in turn provide an understanding of the pathways regulated by NS1 during infection. Therefore, DAVID bioinformatics resources was used to group proteins in the dataset. Several host functions important for virus replication were identified by this analysis (**Table 1**) and protein-interaction networks were derived using STRING database. Cytoscape helped visualize the interactions among the cellular proteins and NS1 in each group (**Figures 3–5**). Thus, bioinformatics analysis of the protein dataset provided an insight into the different host functions that may be regulated through NS1 during virus infection.

We validated our study by confirming the interaction between some of the hnRNP proteins, DDX1 and ILF3. Our IP studies established that hnRNP C, hnRNP K, hnRNP U, ILF3, and DDX1 interacted with the NS1 protein (**Figures 6A–C**). hnRNP K protein is known to interact with the cellular protein NS1-binding protein (NS1-BP) and plays a major role in M1 mRNA splicing by binding directly to M1 mRNA (Tsai et al., 2013). hnRNP U and hnRNP C proteins are differentially expressed in H3N2 virus-infected cells when compared to uninfected cells. Additionally, hnRNP C protein translocates from the nucleus to the cytoplasm and colocalizes with NS1 protein in infected cells (Wu et al., 2013). ILF3 is a component of stress granules and inhibits influenza virus replication through direct interaction with viral NP and PKR (Wang et al., 2009; Wen et al., 2014). DDX1 in complex with other RNA helicases DDX21 and DHX36 acts as a viral sensor to induce type I IFN expression (Zhang et al., 2011). Thus interaction of these cellular proteins with NS1 could have significant implications for influenza virus infection. While we were able to confirm interaction of NS1 with the above mentioned proteins, NS1 did not interact with hnRNPs F, M, and R and ILF2 (**Figures 6C,D**). It is possible that, these proteins form a part of the NS1-interacting complex through its interaction with other NS1-interacting cellular proteins present in the complex. Alternatively, they may also interact with other viral proteins that were identified by LC-MS/MS to be present in the NS1-interacting complex.

Many of the previously known NS1-interacting proteins such as PABP1, DHX9, DDX21, and CRKL were identified in this study. Additionally, 92 out of the 192 proteins analyzed in our dataset were listed as known NS1 interactors in the VirHostNet 2.0 database, which increased the confidence in our dataset (Supplementary Table S1). Meanwhile, some of the well-known NS1 binding partners such as p85β and CPSF30 were not identified in the NS1-interacting protein complex. The absence of these well-known NS1-interacting partners could be due to the inherent limitations of protein identification by mass spectrometry, such as missed and non-specific cleavage of proteins during trypsin treatment and availability of spectral information of proteins in the database (Lubec and Afjehi-Sadat, 2007). Additionally, polymorphisms in the protein sequence of the NS1 used in this study may also be responsible for the absence of the well-known NS1 binding partners. Examples of these polymorphisms playing a role in binding to different cellular partners have been demonstrated in other studies (Shapira et al., 2009; de Chassey et al., 2013).

It is well documented that significant polymorphisms exist in the NS1 sequences among various strains (Marc, 2014). SK02 virus is a wholly avian virus isolated from pigs (Karasin et al., 2004) and because of this the NS1 protein has some unique sequences and motifs. NS1 protein of influenza A viruses are known to have a PDZ-binding domain (PBM) at the last four amino acid residues (Obenauer et al., 2006). As the name suggests, PBMs confer binding to proteins containing a characteristic structure called the PDZ domain. PDZ domain-containing proteins act as scaffolds to assemble protein complexes and function in cell signaling and cell polarity (Harris and Lim, 2001; Nourry et al., 2003). Human influenza A virus isolates contain a PBM with varied sequences depending on the strain and NS1 from H5N1 avian influenza A virus isolates from human infections generally have a PBM with the sequence ESEV (Golebiewski et al., 2011). Some studies have shown that avian NS1 with ESEV PBM can bind PDZ proteins Scribble, Dlg1, MAGI-1, MAGI-2, MAGI-3, and possess an indirect association with Lin7C (Liu et al., 2010; Golebiewski et al., 2011; Thomas et al., 2011). The SK02 virus used in our study for infections has the same avian signature ESEV PBM (AA 227–230) in the NS1 protein. Therefore, we were interested in finding out, whether any of the above mentioned proteins have been identified in our pull-down complex. Consistent with the literature, Dlg1 and Lin7C were indeed present in our dataset (Supplementary Table S2). This interaction has been shown to decrease tight junction integrity of the epithelial cells and benefit virus replication (Liu et al., 2010; Golebiewski et al., 2011; Thomas et al., 2011).

It has been observed that, most avian influenza A viruses have a class II SH3 domain in the NS1 protein with the sequence PPLPPK at AA 212-217, while this motif is rarely seen in human influenza viruses (Heikkinen et al., 2008). NS1 proteins with this sequence have been shown to bind to cellular adaptor protein CRK/CRKL, while the NS1 proteins of human influenza A viruses do not bind to this sequence (Heikkinen et al., 2008; Hrincius et al., 2010). Consequently, NS1-CRK/CRKL interaction inhibits virus induced activation of JNK-ATF2 pathway, which in turn prevents premature cell death and thereby facilitates enhanced virus replication (Hrincius et al., 2010). The NS1 protein of SK02 also possesses a class II SH3 binding motif and CRK/CRKL protein was identified in our pull-down complex (Supplementary Table S1). All these unique interactions could have a significant effect on the pathogenesis of the virus at the cellular level.

Taken together, the cellular proteins identified in this complex could provide an insight into the different pathways important for influenza infection through interaction with NS1 protein. Validation and characterization of these interactions will provide a deeper understanding of the virus–host interplay, the co-evolution mechanisms that molded the host–pathogen relationship and will help in the identification of new therapeutic targets to control influenza infection and disease pathogenesis.

## Conflict of Interest Statement

The authors declare that the research was conducted in the absence of any commercial or financial relationships that could be construed as a potential conflict of interest.

## Footnotes

1 www.matrixscience.com
