# Supplementary material for: Networks of Host Factors that Interact with NS1 Protein of Influenza A Virus
Source: Front Microbiol. 2016 May 4;7:654. doi: 10.3389/fmicb.2016.00654 (PMC4855030; doi:10.3389/fmicb.2016.00654)
Supplement: Supplementary file 1 [file Table_S1.PDF]

List of Proteins above a protein score of 150 in the purified NS1 protein complex

Uniprot ID	Gene Name	Peptides matched	Sequence coverage (%)	Protein Score
Q13085	ACACA	81	47.1	4631
Q00839	HNRNPU	7	12.4	2162
P05166	PCCB	29	73.8	2146
P07437	TUBB5	28	79.3	2031
P05217	TBB2	28	79	1915
P04350	TUBB4A	5	12.3	1903
P68371	TUBB4B	5	12.3	1903
Q9H4B7	TBB1	28	71.2	1861
P39023	RPL3	13	27	1706
Q14573	ITPR3	35	18.1	1606
P62841	RPS15	6	54.9	1508
P11940	PABPC1	29	42.7	1426
P62701	RPS4X	22	61.3	1413
P52272	HNRNPM	16	37.8	1331
P04264	KRT1	16	38.6	1303
Q9H4B7	TUBB1	17	41	1232
P68363	TUBA1B	21	64.7	1198
Q96FU6	ACTG1	21	69.7	1167
P61247	RPS3A	24	67.5	1158
P60709	ACTB	20	64.4	1140
P62753	RPS6	11	32.4	1132
Q9BUF5	TUBB6	17	45.2	1050
P67809	YBX1	12	65.9	1016
Q9BQE3	TUBA1C	20	59.8	1002
P62424	RPL7A	17	48.2	986
Q9Y224	C14orf166	10	39.8	950
Q92499	DDX1	19	32.9	948
P05141	SLC25A5	12	35.9	945
P68366	TUBA4A	15	46.3	939
P36578	RPL4	17	39.3	918
Q8NCA5	FAM98A	10	38.6	906
P52597	HNRNPF	10	36.8	902
P22087	FBL	13	58.4	886
Q12905	ILF2	11	44.2	881
P43243	MATR3	13	27.8	871
P12236	SLC25A6	14	51.6	863
Q6PEY2	TUBA3E	14	49.2	855
Q12906	ILF3	21	35.2	847
Q7Z417	NUFIP2	8	18	846
P62081	RPS7	11	55.2	833
Q13310	PABPC4	18	32.2	806
P68104	EEF1A1	12	47.5	803
P15880	RPS2	16	49.2	795
P12235	SLC25A4	8	30.2	785
Q6UN15	FIP1L1	13	37.7	780
P06576	ATP5B	10	35.2	751
P25705	ATP5A1	10	25.6	746

Uniprot ID	Gene Name	Peptides matched	Sequence coverage (%)	Protein Score
P46776	RPL27A	9	40.3	739
Q02878	RPL6	14	40.3	707
P11498	PC	10	13.4	701
P62241	RPS8	13	54.8	697
P62266	RPS23	6	42.7	667
Q10570	CPSF1	16	16.8	663
P62888	RPL30	7	51.8	661
P14618	PKM	8	26.7	648
P62829	RPL23	6	36	639
P23396	RPS3	17	69.3	639
P62263	RPS14	9	68.3	636
P62249	RPS16	12	52.5	618
P53621	COPA	13	15.8	610
P05165	PCCA	14	22.8	603
Q9Y2T7	YBX2	10	35.7	595
P61978	HNRNPK	9	35.4	587
O00571	DDX3X	8	17.2	572
P13645	KRT10	9	27.6	564
Q07020	RPL18	8	36.4	564
Q02543	RPL18A	9	36.6	564
P62917	RPL8	10	42.5	553
Q9NZB2	FAM120A	10	15.4	547
P27635	RPL10	8	35.6	547
P18124	RPL7	14	46.8	546
P62750	RPL23A	9	44.8	537
P51991	HNRNPA3	7	31.7	527
Q9H361	PABPC3	16	20.4	517
P18621	RPL17	13	51.4	514
O00567	NOP56	8	18.2	503
P11142	HSPA8	14	25.8	499
P26373	RPL13	13	44.6	488
P62277	RPS13	13	49.8	488
Q12959	DLG1	9	18	487
Q3ZCM7	TUBB8	10	22.1	473
P35527	KRT9	7	28.8	472
P62244	RPS15A	10	61.1	461
Q96CW1	AP2M1	11	34.6	454
P16402	HIST1H1D	7	23.1	450
P62269	RPS18	10	42.2	443
P04406	GAPDH	7	33.3	434
P62805	HIST1H4A	7	52.3	411
P55795	HNRNPH2	8	30.9	409
Q5SSJ5	HP1BP3	13	28.9	409
Q08211	DHX9	12	11	407
P14866	HNRNPL	6	23.5	407
P06899	HIST1H2BJ	5	38	391
P46782	RPS5	4	36.3	391

Uniprot ID	Gene Name	Peptides matched	Sequence coverage (%)	Protein Score
Q02978	SLC25A11	5	30.5	391
P07910	HNRNPC	5	19.5	381
P62913	RPL11	7	36.5	376
P62979	RPS27A	3	19.8	371
Q92974	ARHGEF2	8	13.3	367
P84098	RPL19	5	21	367
P46778	RPL21	9	37.2	361
Q07065	CKAP4	8	17.3	357
P62847	RPS24	6	31.1	353
Q9Y2X3	NOP58	4	11.9	349
O60506	SYNCRIP	6	12.6	346
P26599	PTBP1	4	18.3	342
O43390	HNRNPR	7	12.4	337
Q9Y6M1	IGF2BP2	7	16.4	337
P16401	HIST1H1B	6	18.5	329
P62280	RPS11	9	46.7	327
Q9C0J8	WDR33	12	16	326
P10412	HIST1H1E	4	12.5	324
P83731	RPL24	5	25.8	323
P05455	SSB	5	11	322
P46781	RPS9	12	31.1	319
Q92841	DDX17	8	21	313
P61313	RPL15	10	36.7	313
Q3KQU3	MAP7D1	5	6.8	300
Q8WWM7	ATXN2L	13	14.4	287
Q13435	SF3B2	6	12	277
P22492	HIST1H1T	3	8	276
O15523	DDX3Y	5	10.6	272
Q9BQG0	MYBBP1A	6	8.1	272
P62910	RPL32	5	33	270
Q9HCE1	MOV10	3	3.5	269
P08106	HSPA2	6	12.7	266
Q9NR30	DDX21	7	12	260
Q1KMD3	HNRNPUL2	7	9.8	258
P50914	RPL14	5	21.6	258
P62899	RPL31	7	44.9	258
P16104	H2AFX	4	37.8	256
Q14204	DYNC1H1	9	2.9	254
P62158	CALM1	3	49	252
P27482	CALML3	3	49	252
Q6NZI2	PTRF	6	20	252
P23528	CFL1	5	47.4	244
P45880	VDAC2	3	18.5	241
Q15717	ELAVL1	4	24.9	240
P68431	HIST1H3A	2	29.1	239
P0DMV9	HSPA1B	6	13.3	238
P04792	HSPB1	4	38.8	236
O60884	DNAJA2	4	18.8	234

Uniprot ID	Gene Name	Peptides matched	Sequence coverage (%)	Protein Score
P51116	FXR2	5	11.3	232
P62851	RPS25	4	23.9	228
P84103	SRSF3	4	26.5	225
Q13084	MRPL28	4	27.3	224
P47914	RPL29	3	18.8	224
P42766	RPL35	4	24.9	223
O76094	SRP72	8	18.9	223
Q9P2I0	CPSF2	4	7.1	222
P46109	CRKL	9	35.6	218
P02511	CRYAB	5	51.9	218
P17858	PFKL	6	16.2	218
P36542	ATP5C1	4	15.9	216
P62854	RPS26	3	42.2	210
Q13405	MRPL49	2	14.9	207
O43143	DHX15	5	10.1	206
Q9BUJ2	HNRNPUL1	4	10.3	200
O95793	STAU1	6	12.1	197
Q96A33	CCDC47	3	10.4	196
Q13509	TUBB3	5	15.1	195
P61513	RPL37A	4	40.9	193
P51114	FXR1	7	18.4	190
P11387	TOP1	5	7.5	190
P83881	RPL36A	4	25.9	186
Q9Y3U8	RPL36	6	36.7	185
Q00325	SLC25A3	4	16.6	183
P38159	RBMX	6	15.9	181
Q8TCJ2	STT3B	4	6.9	179
P84243	H3F3A	2	29.3	174
Q6PKG0	LARP1	4	3.9	173
P61254	RPL26	8	32.5	173
Q8WXX5	DNAJC9	2	11.7	172
Q9Y3Y2	CHTOP	3	17.5	171
O14910	LIN7A	3	15.6	171
Q9NUP9	LIN7C	5	33.6	171
P52815	MRPL12	2	12.8	171
P49411	TUFM	6	17.3	169
P32969	RPL9	2	15	166
P40429	RPL13A	8	30.3	165
P07305	H1F0	3	18.4	162
P42677	RPS27	5	38.2	162
P0DMV8	HSPA1A	3	7.4	158
P08107	HSPA1A	6	13.3	158
Q86V81	ALYREF	5	40	157
Q86V81	THOC4	5	40	157
Q99459	CDC5L	2	3.2	156
P67936	TPM4	4	18.1	155
Q9UJS0	SLC25A13	2	5.2	152
Q99729	HNRNPAB	3	11	151

Uniprot ID	Gene Name	Peptides matched	Sequence coverage (%)	Protein Score
Q9H0A0	NAT10	3	4.1	151
Q13283	G3BP1	2	7.4	150

Table S1. List of proteins above a protein score of 150 in the purified NS1 protein complex. The table represents the list of all proteins in the purified protein complex, identified using mass spectrometry with a protein score of 150 and above. The proteins documented in the VirHostNet 2.0 database as known NS1 interacting partners is highlighted with a dark tan colour.
