# Supplementary material for: Networks of Host Factors that Interact with NS1 Protein of Influenza A Virus
Source: Front Microbiol. 2016 May 4;7:654. doi: 10.3389/fmicb.2016.00654 (PMC4855030; doi:10.3389/fmicb.2016.00654)
Supplement: Supplementary file 2 [file Table_S2.PDF]

Translation

Uniprot ID	Gene Name
P49411	TUFM
P68104	EEF1A1
P27635	RPL10
P62913	RPL11
P26373	RPL13
P40429	RPL13A
P50914	RPL14
P61313	RPL15
P18621	RPL17
Q07020	RPL18
Q02543	RPL18A
P84098	RPL19
P46778	RPL21
P62750	RPL23A
P83731	RPL24
P61254	RPL26
P46776	RPL27A
P46779	RPL28
P47914	RPL29
P62888	RPL30
P62899	RPL31
P42766	RPL35
Q9Y3U8	RPL36
P83881	RPL36A
P61513	RPL37A
P39023	RPL3
P36578	RPL4
Q02878	RPL6
P62424	RPL7A
P62917	RPL8
P32969	RPL9
P46783	RPS10
P62280	RPS11
P62277	RPS13
P62263	RPS14
P62841	RPS15
P62244	RPS15A
P62249	RPS16
P62269	RPS18
P15880	RPS2
P62266	RPS23
P62847	RPS24
P62851	RPS25
P42677	RPS27
P62979	RPS27A
P23396	RPS3
P61247	RPS3A
P62701	RPS4X
P46782	RPS5
P62753	RPS6
P62081	RPS7
P62241	RPS8
P46781	RPS9
P62910	RPL32
P52815	MRPL12
Q13084	MRPL28
Q13405	MRPL49
Q13310	PABPC4
P40429	RPL13A

RNA Processing

Uniprot ID	Gene Name
Q99459	CDC5L
Q92499	DDX1
Q92841	DDX17
O43143	DHX15
Q08211	DHX9
Q6UN15	FIP1L1
Q9HCE1	MOV10
O00567	NOP56
Q9Y2X3	NOP58
P05455	SSB
Q86V81	THOC4
P67809	YBX1
Q10570	CPSF1
Q9P2I0	CPSF2
P22087	FBL
P51991	HNRNPA3
P07910	HNRNPC
P52597	HNRNPF
P61978	HNRNPK
P52272	HNRNPM
O43390	HNRNPR
Q00839	HNRNPU
Q9BUJ2	HNRNPUL1
Q13310	PABPC4
P11940	PABPC1
P26599	PTBP1
P62913	RPL11
P50914	RPL14
P61254	RPL26
P83881	RPL36A
P62263	RPS14
P62841	RPS15
P62249	RPS16
P62847	RPS24
P62753	RPS6
P62081	RPS7
P38159	RBMX
Q13435	SF3B2
P84103	SRSF3
O60506	SYNCRIP
P14866	HNRNPL

Splicing

Uniprot ID	Gene Name
Q99459	CDC5L
Q92499	DDX1
O43143	DHX15
Q08211	DHX9
Q86V81	THOC4
P67809	YBX1
Q10570	CPSF1
Q9P2I0	CPSF2
P51991	HNRNPA3
P07910	HNRNPC
P52597	HNRNPF
P61978	HNRNPK
P52272	HNRNPM
O43390	HNRNPR
Q00839	HNRNPU
Q9BUJ2	HNRNPUL1
P11940	PABPC1
P26599	PTBP1
P83881	RPL36A
P38159	RBMX
P14866	HNRNPL
Q13435	SF3B2
P84103	SRSF3
O60506	SYNCRIP

Microtubule & Cytoskeleton

Uniprot ID	Gene Name
Q08211	DHX9
Q3KQU3	MAP7D1
Q92974	ARHGEF2
P62158	CALM1
Q9Y224	C14orf166
P02511	CRYAB
Q14204	DYNC1H1
P04792	HSPB1
P68363	TUBA1B
O95793	STAU1
Q9BQE3	TUBA1C
Q6PEY2	TUBA3E
P68366	TUBA4A
Q9H4B7	TBB1
P05217	TBB2
Q13509	TUBB3
P04350	TUBB4A
Q9BUF5	TUBB6
Q3ZCM7	TUBB8
P07437	TUBB5

Apoptosis

Uniprot ID	Gene Name
P23528	CFL1
P02511	CRYAB
P04792	HSPB1
P08107	HSPA1A
P62979	RPS27A
P16989	YBX3
P23396	RPS3
P61247	RPS3A
P05217	TBB2
P07437	TUBB5
Q92974	ARHGEF2
P62913	RPL11
P62753	RPS6

PDZ domain containing

Uniprot ID	Gene Name
Q12959	DLG1
O14910	LIN7A
Q9NUP9	LIN7C

Innate Immunity

Uniprot ID	Gene Name
P62158	CALM1
P46109	CRKL
P02511	CRYAB
Q92499	DDX1
Q9NR30	DDX21
O00571	DDX3X
Q08211	DHX9
Q15717	ELAVL1
P51114	FXR1
P14866	HNRNPL
Q12906	ILF3
Q14573	ITPR3
Q9HCE1	MOV10
Q15365	PCBP1
Q15366	PCBP2
P84098	RPL19
P49411	TUFM
P67809	YBX1

Table S2. Enriched functions as analyzed by DAVID resources and InnateDB. This table represents the different enriched functional categories identified using DAVID bioinformatics resources and contains the protein belonging to each functional category. Proteins related to innate immunity were grouped using InnateDB databse.
